# Generation of immunodeficient pig with hereditary tyrosinemia type 1 and their preliminary application for humanized liver

**DOI:** 10.1186/s13578-022-00760-3

**Published:** 2022-03-07

**Authors:** Jilong Ren, Dawei Yu, Jing Wang, Kai Xu, Yanan Xu, Renren Sun, Peipei An, Chongyang Li, Guihai Feng, Ying Zhang, Xiangpeng Dai, Hongye Zhao, Zhengzhu Wang, Zhiqiang Han, Haibo Zhu, Yuchun Ding, Xiaoyan You, Xueqin Liu, Meng Wu, Lin Luo, Ziyi Li, Yong-Guang Yang, Zheng Hu, Hong-jiang Wei, Liangpeng Ge, Tang Hai, Wei Li

**Affiliations:** 1 State Key Laboratory of Stem Cell and Reproductive Biology, Institute of Zoology, Chinese Academy of Sciences, Beijing, 100101 China; 2 Institute for Stem Cell and Regenerative Medicine, Chinese Academy of Sciences, Beijing, 100101 China; 3 Beijing Institute for Stem Cell and Regenerative Medicine, Beijing, 100101 China; 4 Beijing Farm Animal Research Center, Institute of Zoology, Chinese Academy of Sciences, Beijing, 100101 China; 5 Key Laboratory of Organ Regeneration and Transplantation of the Ministry of Education, First Hospital, Jilin University, Changchun, 130062 China; 6 Institute of Animal Sciences (IAS), Chinese Academy of Agricultural Sciences (CAAS), Beijing, 100193 China; 7 University of Chinese Academy of Sciences, Beijing, 100049 China; 8 Chongqing Academy of Animal Sciences, Chongqing, 402460 China; 9 Key Laboratory of Pig Industry Sciences, Ministry of Agriculture, Chongqing, 402460 China; 10 Chongqing Key Laboratory of Pig Industry Sciences, Chongqing, 402460 China; 11 Technical Engineering Center for the Development and Utilization of Medical Animal Resources, Chongqing, 402460 China; 12 State Key Laboratory for Conservation and Utilization of Bio-Resources in Yunnan, Yunnan Agricultural University, Kunming, 650201 China; 13 Center of Reproductive Medicine and Center of Prenatal Diagnosis, First Hospital, Jilin University, Changchun, 130021 China

**Keywords:** Liver regeneration, Immunodeficiency, Gene editing, Disease model

## Abstract

**Background:**

Mice with humanized livers are important models to study drug toxicology testing, development of hepatitis virus treatments, and hepatocyte transplantation therapy. However, the huge difference between mouse and human in size and anatomy limited the application of humanized mice in investigating human diseases. Therefore, it is urgent to construct humanized livers in pigs to precisely investigate hepatocyte regeneration and human hepatocyte therapy. CRISPR/Cas9 system and somatic cell cloning technology were used to generate two pig models with *FAH* deficiency and exhibiting severe immunodeficiency (*FAH/RAG1* and *FAH/RAG1/IL2RG* deficiency). Human primary hepatocytes were then successfully transplanted into the *FG* pig model and constructed two pigs with human liver.

**Results:**

The constructed *FAH/RAG1/IL2RG* triple-knockout pig models were characterized by chronic liver injury and severe immunodeficiency. Importantly, the *FG* pigs transplanted with primary human hepatocytes produced human albumin in a time dependent manner as early as 1 week after transplantation. Furthermore, the colonization of human hepatocytes was confirmed by immunochemistry staining.

**Conclusions:**

We successfully generated pig models with severe immunodeficiency that could construct human liver tissues.

**Supplementary Information:**

The online version contains supplementary material available at 10.1186/s13578-022-00760-3.

## Introduction

Liver can regenerate itself after injury. Regeneration of human livers in animal models can yield a substantial number of human hepatocytes, which could be used for subsequent liver transplantation [7, 17, 18, 34]. Replacement of diseased mouse liver by allogenic hepatocyte transplantation has been shown to be feasible in a urokinase-type plasminogen-activator (*uPA*) transgenic mouse model [29]. The transplanted woodchuck hepatocytes could reconstitute up to 90% of the *uPA* with depletion of the recombination activating gene 2 (*RAG2*) in mouse liver [27]. Using the same model and a similar method, human hepatocytes were estimated to constitute up to 15% in the *uPA/RAG2* mouse liver [5]. Further study indicated that human hepatocytes repopulated in the *uPA*^+/+^-SCID mouse liver, preserved normal HBV-infected function [20]. Depletion of fumarylacetoacetate hydrolase (*FAH*), an enzyme that catalyzes the last step of tyrosine metabolism, led to a hereditary tyrosinemia type I (HT1). *FAH*-deficiency caused a lethal defect in utero in human and after birth in animal model of mice. The defect can be corrected by administration of 2-(2-nitro-4-trifluoromethylbenzoyl)-1,3 cyclohexanedione (NTBC). The humanized liver mouse model with *FAH* deficiency provided a reliable model of liver repopulation using transplanted cells [8, 23, 24]. The triple-knockout (KO) mouse model (*FRG*), generated by crossing double KO *FAH*^*−/−*^*RAG2*^*−/−*^ mice with *IL2RG*^*−/−*^ mice with deficiency in the common γ-chain of the interleukin receptor, can be efficiently repopulated with human hepatocytes [3]. The animals pretreated by urokinase-expressing adenovirus showed a high capability for engraft (up to 90%) with human hepatocytes from multiple sources [1, 9, 11, 15].

Due to small body size of mice, it is difficult to obtain large numbers of human hepatocytes in the mouse models. Therefore, it is necessary to generate a large animal model for humanized liver, and intrauterine methods were employed to solve immune-related problems. Piglets postnatally engrafted with human hepatocytes produced significant levels of human albumin in their serum, but the efficiency was far to satisfied [22]. *FAH* pigs were also produced [12, 14]. Besides, *RAG1*-knockout pigs were confirmed to lack mature T-cells and B-cells, but contained a substantial number of cells which appeared to be T-cell or B-cell progenitors caused by animal T/B cell developmental disorder as well as NK-cells [16, 19, 31].

In this study, we characterized the humanized liver pigs generated based on the severely immunodeficient *FAH*-deficient cloned pigs using CRISPR/CRISPR-associated protein 9 (Cas9) and somatic-cell nuclear transplantation (SCNT). The *FRG* pigs transplanted with primary human hepatocytes produced human albumin and exhibited colonization of human hepatocytes confirmed by immunochemistry staining. Therefore, our pig models offer valuable evidences for the usage of pigs in the field of hepatocyte transplantation and liver regeneration.

## Results

### Generation of ***FAH***^***−/−***^***IL2RG***^***−/Y***^(***FG***) pigs

**Fig. 1:**
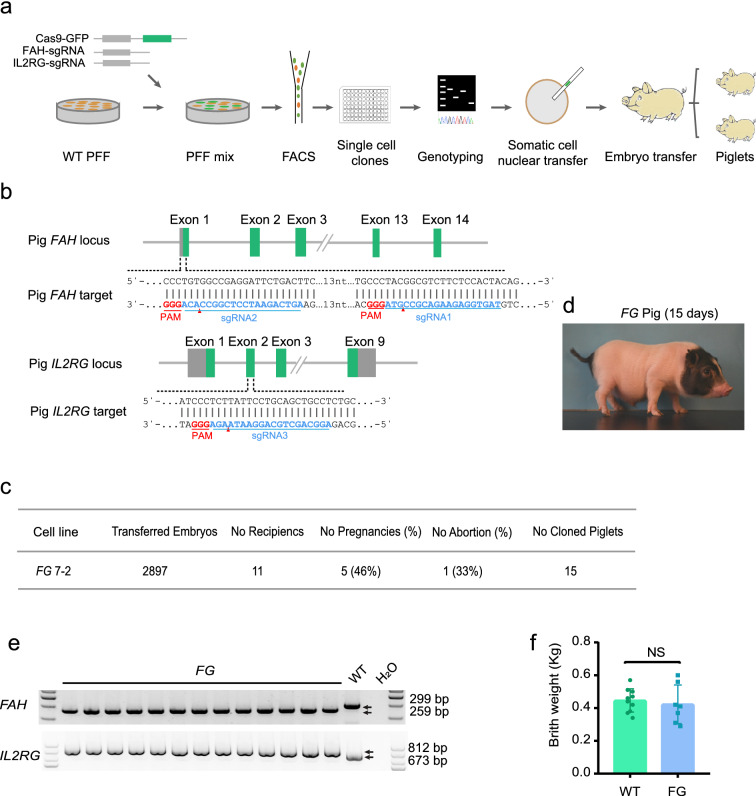
**a** Experimental procedures for FG pig model generation. **b** Schematic diagram of sgRNA-targeting exons of porcine FAH and IL2RG. **c** Summary of embryo transfer data from SCNT of FG knockout cell-line to generate mutant pig models. **d** Appearance of a 15-day-old FG model pig. **e** The birth weight between model and wild-type piglets. **f** Genomic PCR results of the FAH/ IL2RG knockout pig.

The *FG* pig models were generated by CRISPR/Cas9 system and SCNT techniques. The methods used to construct the animal models are illustrated in Fig. 1a. Briefly, the sgRNAs targeting pig *FAH* and *IL2RG* were designed and their cutting efficiencies were tested (Fig. 1b). The sgRNA sequences with high cutting efficiency were selected for targeting each gene (Fig. 1b). The PFF of Bama miniature pigs were then transfected by electroporation with the appropriate vectors. More than ten *FG* single cell colonies were obtained following flow cytometry filtering. Sanger sequencing was used to genotype the cell colonies and some of the genotyping results are listed in Additional file 1: Fig. S1. The cell line *FG*7-2 was used to generate 2,897 cloned embryos, which were then transferred into 11 surrogates. After 28–32 days, five surrogates were detected pregnancy by B-ultrasound, one of which aborted in late pregnancy. Finally, 15 piglets were born from four surrogates (Fig. 1c). *FG* piglets were kept in a clean conventional housing environment (Fig. 1d). At birth, the umbilical cord tissues of each piglet were collected and genotyped. DNA sequencing results revealed that all piglets were consistent with their corresponding cell-line genotype (Fig. 1e). Furthermore, the birth weight of piglets was measured and there was no significant difference between *FG* and wild-type piglets (Fig. 1f).

### Phenotypic identification of *FG* pig models

**Fig. 2:**
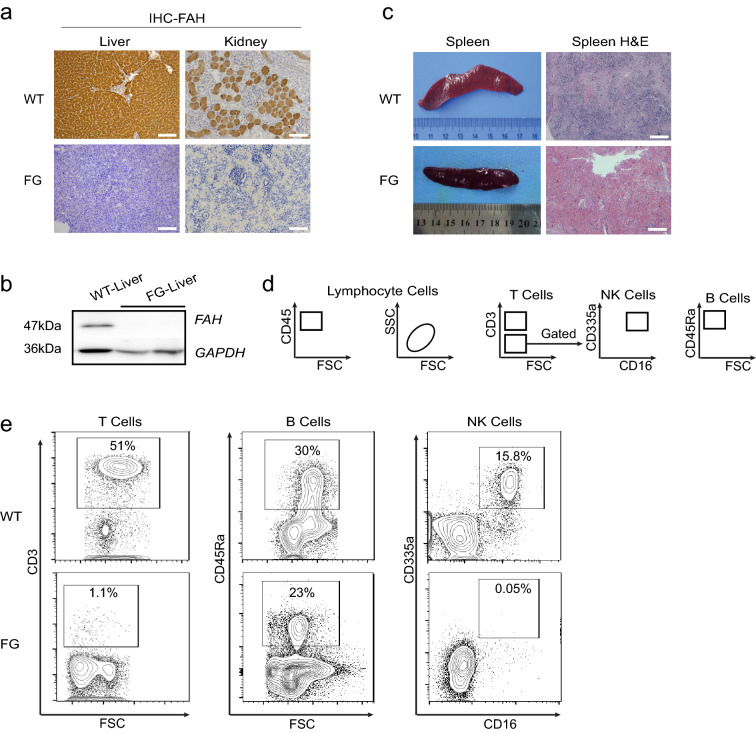
Phenotypic analysis of *FG* pigs. **a** Immunohistochemical staining of wild-type and *FG* pig liver and kidney tissues with *FAH* antibodies. Scale bar = 100 μm. **b** Western blotting results showed that *FAH* protein is missing in the liver tissue of *FG* model pigs. **c** HE staining of the WT and *FRG* pig spleen tissue. Scale bar = 200 μm. **d** Schematic diagram of separation and analysis of immune cells compositions in the pig peripheral blood. **e** Flow cytometric analysis of T-cells, B-cells, and NK-cells in the peripheral blood of *FG*-knockout pigs

To further confirm the phenotype of the *FG* pigs, liver, kidney, and other tissues of piglets were dissected for pathological examination (Fig. 2). The liver and kidney tissue were subjected to immunohistochemical staining and western blotting with *FAH* antibody. No signal can be detected by *FAH* antibody in either model (Fig. 2a, c). The immune system tissues, thymus and spleen of all *FG* piglets were dissected at the endpoint for further immunological phenotype characterization. Six of fifteen *FG* pigs had no thymus and the remaining nine *FG* pigs displayed severe thymus atrophies phenotype. The model pigs were absent for spleen tissue structure and showed fewer lymphocytes than that of normal pigs (Fig. 2b). Peripheral blood collected from the model pigs was analyzed by flow cytometry (Fig. 2d and e). In detail, the proportion of CD3-positive T cells in the wild-type pigs was 51%, but was only 1.1% in *FG* model pigs. The proportion of CD16 and CD335a double-positive NK cells in the wild-type pigs was 15.8%, while that in *FG* model pigs was only 0.05%. A combined analysis of CD3-negative and CD45RA-positive cells in the peripheral blood was performed and the results showed a proportion of 30% in wild-type and 23% in *FG* pigs. Therefore, we observed an absence of T cells and NK cells in *FG* model pigs which is consistent with previous reports. Furthermore, the basic blood routine and blood biochemistry between the pig models was analyzed (Additional file 3: Tables S1 and S2).

### Human hepatocyte transplantation in *FG* pigs

**Fig. 3:**
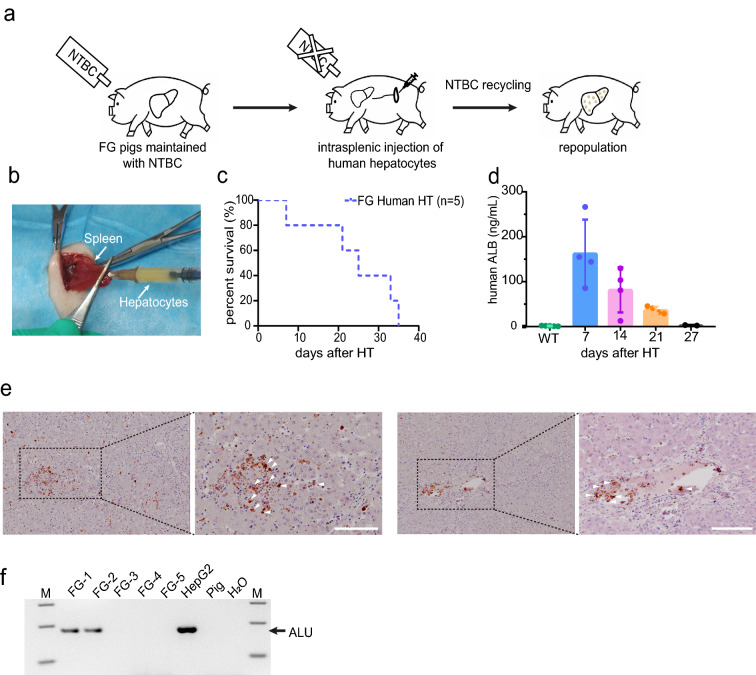
Preliminary results of human hepatocyte transplantation in *FG* pigs. **a** Diagrammatic sketch of human hepatocyte transplantation. **b** Surgical injection of human hepatocytes into pig spleen. **c** Survival curve of pigs after human hepatocyte transplantation. **d** Changes of human albumin concentration in pig peripheral blood following human hepatocyte transplantation. **e** Detection of human hepatocytes in the liver sections of pigs after death. **f** PCR detection of human ALU gene in pig liver tissue after human hepatocyte transplantation

Five healthy *FG* pigs were selected for human hepatocyte transplantation to generate humanized livers in them. The transplantation process were performed as previously described [2] (Fig. 3a). Human hepatocytes (1 × 10^7^/mL) were transplanted into the spleen tissue of *FG* pigs at 3–5 days after birth (Fig. 3b). However, one of the piglets died 3 days following hepatocyte transplantation, and the remaining piglets survived for more than 21 days. The survival curves after transplantation are shown in Fig. 3c. To evaluate the survival and regeneration of human hepatocytes in the pigs, peripheral blood was collected from the piglets every 7 days. In the first 7 days, human albumin was detected in the peripheral blood of all four piglets and their concentrations were ranging from 85.4 to 266.3 ng/µL. On the fourteenth day after transplantation, this concentration of human albumin was decreased to 12.7–129.6 ng/mL. At the day of 21, human albumin in the peripheral blood was only 28.8–45.5 ng/mL. The concentration of human albumin was further decreased to 2–3 ng mL^−1^ at day 28 after transplantation (Fig. 3d). Importantly, *FAH-*positive cells can be detected in the hilum and blood vessels by immunohistochemical staining on the liver tissue with *FAH* antibody which indicating that a small number of human hepatocytes had entered and implanted in the liver of the two *FG* pigs (Fig. 3e). Pig liver tissues transplanted with human hepatocytes were subjected to PCR analysis and the presence of human-specific genes was confirmed by the identification of human DNA (Fig. 3f).

### Generation of *FAH*^*−/−*^*RAG*^*−/−*^*IL2RG*^*−/Y*^(*FRG*) pigs

**Fig. 4:**
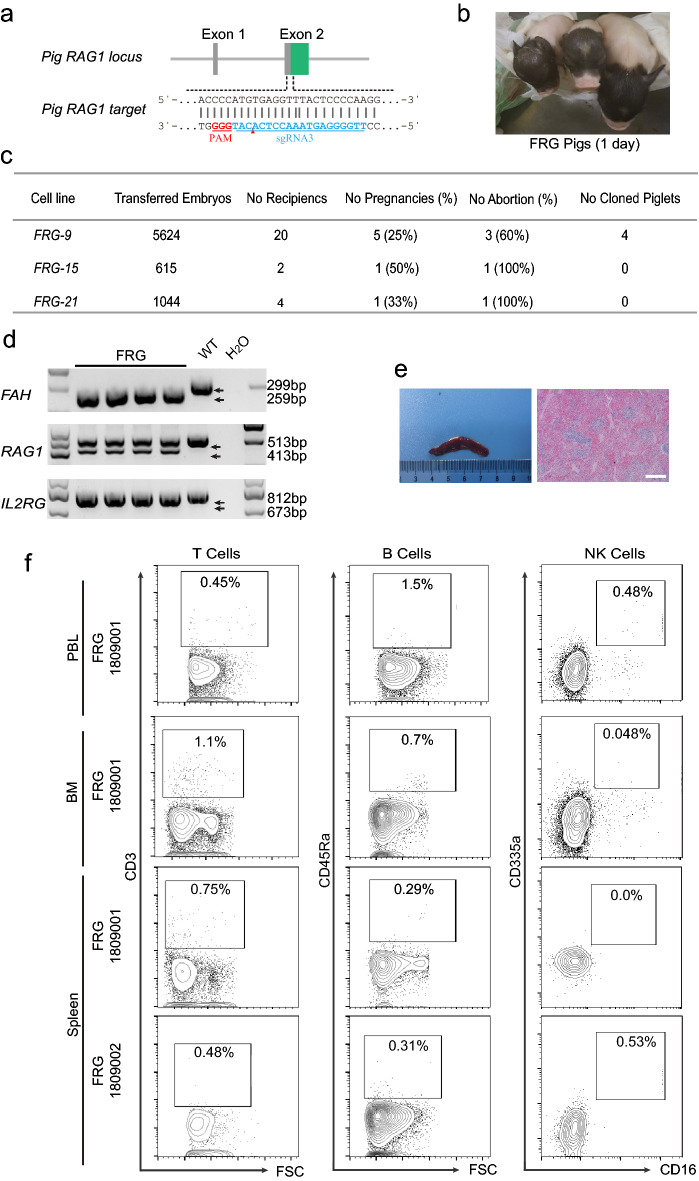
**a** sgRNA-targeted exon of porcine RAG1. The PAM and target sequences are colored in red and blue, respectively, and the cutting sites are indicated by small red triangles. **b** Appearance of the FRG model pigs. **c** Summary of embryo transfer data from SCNT of FRG knockout cell-line to generate mutant pig models. **d** Genomic PCR results confirming the pig FAH/RAG1/IL2RG knockout. **e** HE staining of the WT and FRG pig spleen tissue. Scale bar = 200 μm. **f** Flow cytometric analysis of T cells, B cells, and NK cells in the peripheral blood, bone marrow and spleen of the FRG pig.

Furthermore, the *FRG* triple-knockout pig models were generated by the same method used in establishing *FG* pig models. The strategy of *RAG1* and *FRG* genes knockout is shown in Fig. 4a and 4b, respectively. Three cell lines with best growth status, named FRG-9, FRG-15 and FRG-20, were selected from the 16 screened cell lines with correct genotype. 5,624, 615, and 1,044 embryos were constructed from these cell lines, and were transplanted into 20, 2 and 4 surrogates, respectively. However, only two surrogates which were transplanted with FRG-9 cell constructed embryos maintained pregnancy to term, and finally gave birth to four piglets (Fig. 4c). DNA analysis results of umbilical cord tissues indicated that their genotypes were consistent with the donor cell line which confirmed both of the pigs were *FRG* triple-knockouts (Fig. 4d). Importantly, their spleens were significantly atrophied and H&E staining results also showed that the spleen were not developed completely (Fig. 4e). Consistently, the peripheral blood, spleen and bone marrow of *FRG* piglets were further analyzed by FACS and the T, B, or NK cells in these tissues were rarely detected (Fig. 4f). However, the body weight at birth of *FRG* pigs was significantly lower than that of the *FG* and wild-type pigs (Additional file 2: Fig. S2a). Although these *FRG* pigs were reared in a bacteria-free environment, the longest survival time for the four piglets was only 13 days (Additional file 2: Fig. S2) for some undefined reasons which need further in deep investigation.

## Discussion

It was reported that *FAH*^*−/−*^*RAG2*^*−/−*^*IL2RG*^*−/Y*^ (*FRG*) mice can be used to efficiently repopulated human hepatocytes [1]. This model is widely used in toxicological testing of drug metabolites and exploring experimental gene- and cell-based therapies [13, 30, 33]. However, given their smaller body size, *FRG* mice only produce a relatively low number of human hepatocytes. Porcine liver is a realistic alternative model for human liver regeneration because their organ size and anatomical structure which are more resemble to human [21]. This similarity in size and anatomy between porcine and human benefits the evaluation of cell therapy techniques and their adverse effects [23]. Therefore, *FRG* pigs may be utilized as bioreactors for large-scale production of human hepatocytes and as liver donors for human.

In this study, we successfully generated a severely immunodeficient *FAH*^*−/−*^ pigs which survived for 3 days to 6 weeks in conventional settings. *FG* pig transplanted with human hepatocyte exhibited the highest expression of human albumin at 1 week after transplantation. The human albumin concentration gradually decreased, possibly because (1) a proportion of B-lymphocytes were remained in the *FG* model pigs. (2) The shorter survival time of the pig leave no enough time for growth of the human liver cells. (3) The number of transplanted human hepatocytes (10^7^) is a relatively low cell volume for pig. It was reported that, to achieve a best efficiency, 350 to 864 million live cells are required when performing allogeneic hepatocyte transplantation in pigs [13].

Furthermore, to set up a suitable environment for human hepatocyte colonization, any residual immune cells in model pigs should be removed using antibodies or other methods before the transplantation of human hepatocytes. Alternatively, we may also need to first construct a humanized immune system in pigs and then perform human hepatocyte transplantation which will improve the survivability of human hepatocyte in the porcine model. A study that described an experiment for the transplantation of human hepatocytes into the *RAG2/FAH* double knockout pigs, indicated that immature human hepatocytes successfully engrafted in *FR* swine after IUCT. But the presented NK cells are significant barrier to the expansion of hepatocytes [22]. This report provides us with some new methods and ideas to optimize our model.

The environment in which the pigs live, might be an important factor for the shorter survival of the model pigs. The *FRG* pigs survived for only a short period in the SPF environment in which period the pigs lack enough time to facilitate transplantation of the primary human hepatocytes which only can be detected in a short time. Many researchers [4] encountered problems for the survival of immunodeficient pigs, which highlighting the importance of the improvement of rearing environment. Furthermore, some SCNT-produced animals may be subject to early death, due to incomplete reprogramming during early embryonic development, or other problems caused by cloning techniques itself [10, 25, 26]. However, currently *FRG* pigs could survive for more than 5 months in our optimized conditions (data not shown).

Altogether, we generated a porcine model with colonized human hepatocytes which can produce the human albumin. Our research could offers a useful experimental evidence to further improve the method of generation of such pig models in a high colonization efficiency for human hepatocytes.

## Materials and methods

### Animals and ethics statement

All pigs were raised under the Guidelines for the Care and Use of Laboratory Animals Committee of the Institute of Zoology, Chinese Academy of Sciences. Pigs were raised at the Beijing Farm Animal Research Center.

### Design and construction of the sgRNAs

Single-guide RNAs (sgRNAs) targeting the relevant porcine genes were designed online (http://crispor.tefor.net/). sgRNA oligonucleotide sequences complementary to the *FAH*, *IL2RG*, and *RAG1* genes were annealed and cloned into the BsaI site of the U6-sgRNA vector. The U6-sgRNA and Cas9-eGFP vectors were gifts of Qi Zhou (Institute of Zoology, Chinese Academy of Sciences).

### Generation of *FAH*^−/−^*IL2RG*^*−/Y*^* and FAH*^−/−^*RAG1*^*−/−*^*IL2RG*^*−/Y*^ PFF cell lines

The porcine fetal fibroblast (PFF) cells were isolated and cultured as described previously [6]. Cas9-GFP and sgRNA plasmids were then co-transfected into PFF cells using the 4D-Nucleofector™ system (Lonza, Germany). The transfected cells were harvested after 48 h, and single cell were prepared via flow cytometry, and cultured for 7–10 days in DMEM (Gibco, USA) supplemented with 15% fetal bovine serum (Gibco, USA) at 37 °C, 5% CO_2_. The culture medium was replaced every 3 days. PCR and sequencing analyses were used to determine the propagation and genotypes of the single cell colonies.

### Oocytes maturation, SCNT and embryo transfer

Pig ovaries were collected from local slaughterhouses and kept in 0.9% NaCl supplemented with 200 IU/mL penicillin and streptomycin at 35–37 °C and transported to the laboratory. Cumulus oocyte complexes (COCs) were aspirated from ovarian follicles using an 18-gauge needle connected to a 10 mL syringe. The collected cumulus-oocyte complexes (COCs) were rinsed three times by HEPES-buffered Tyrode’s medium with 0.01% PVA. Groups of 50 COCs were cultured at 39 °C, 5% CO_2_ for 42–44 h in the wells of 24-well culture plates for maturation and each well contained 500 μL in vitro maturation medium and 400 μL mineral oil. The cumulus cells were removed using 0.1% hyaluronidase in HEPES-buffered Tyrode’s medium. SCNT was performed as described previously [28]. Briefly, matured oocytes and PFFs were placed into manipulation medium supplemented with 7.5 mg/mL cytochalasin B. After enucleation, PFFs were placed into the perivitelline space. The reconstructed embryos were fused in fusion medium using two direct pulses of 1.2 kV cm^−1^ each for 30 microseconds. The reconstructed embryos were cultured in 500 μL porcine zygote medium 3 (PZM3) at 5% CO_2_, 38 °C for 20–24 h prior to embryos transfer. Embryos were surgically transferred into the oviduct of a surrogate the day after estrus was observed. Pregnancy was diagnosed 30 days after embryo transfer. At the day of birth, cloned piglets were natural birth or removed for the surrogates by caesarean section in bacteria free equipment.

### Genotyping of *FG and FRG* piglets

The ear tips of newborn fetuses and piglets were collected into 1.5-mL centrifuge tubes. MicroElute® Genomic DNA Kits (Omega, USA) were used to extract genomic DNA. DNA samples were analyzed using PCR with specific primers for the *FAH*, *RAG1*, or *IL2RG* genes. The PCR reaction program was: 95 °C for 5 min, 35 cycles of 95 °C for 30 s, 60 °C for 30 s, and 72 °C for 45 s and finally 72 °C for 10 min. Primers are listed in Additional file 3: Table S2. The PCR product (5 µL) was subjected to 2% agarose gel electrophoresis in the presence of ethidium bromide solution, and visualized using the iBright CL1000 imaging system (Invitrogen, USA).

### Western blotting

Adipose tissue was dissected and frozen immediately in liquid nitrogen, and stored at − 80 °C until further usage. Total protein was extracted from the tissue samples using RIPA lysis and extraction buffer (Thermo Scientific, USA). Anti-FAH antibody (ab83770) and anti-GAPDH antibody (ab131602) were purchased from Abcam. Equal amounts of tissue sample lysates were resolved by SDS-PAGE and immunoblotted with indicated antibodies. The blots were developed using HRP-conjugated secondary antibodies and an ECL Plus system. All signals were visualized and analyzed using the iBright CL1000 imaging system (Invitrogen, USA).

### Immunohistochemical staining

Dissected tissues were fixed in 10% neutral formalin solution (Sangon Biotech, China) for 24–36 h, then were embedded in paraffin and further sectioned, and stained with hematoxylin and eosin (Thermo, USA). Liver samples were prepared according to the protocol previously reported [35], and immunohistochemical analysis was performed using SP Rabbit & Mouse HRP Kits (CWBIO, China).

### Flow cytometry

Cell suspensions of spleen and bone marrow for *FG*/*FRG*-knockout pigs and age-matched control pigs were prepared. Porcine peripheral blood lymphocyte isolation kits (Solarbio, China) were used to enrich lymphocytes from blood. The erythrocytes in the spleen and bone marrow were removed by erythrocyte lysate (BD, USA). To identify the porcine CD3^+^ T-cells, CD45Ra^+^CD3^−^B-cells, and CD16^+^CD335a^+^CD3^−^NK-cells [32], samples were analyzed by MoFlo XDP (Beckman Coulter, USA).

### Intrasplenic transplantation of primary human hepatocytes

Cryopreserved human hepatocytes were provided by Celsis In Vitro Technologies (Baltimore, MD, USA) and the information of this cell donor is listed in detail in Additional file 3: Table S1. The cryopreserved primary human hepatocytes were suspended in DMEM supplemented with 10% fetal bovine serum, and trypan blue was used to quantify cell viability and the percentage of viable cells are usually more than 90%. Human hepatocytes were suspended in normal saline and injected into the spleen of 3 to 5-day-old FG pigs [35].

### ELISA for detecting human albumin

Tubes containing EDTA as an anticoagulant were used to collect the peripheral blood from pigs. Serum was centrifuged at 2000*g*. The concentration of human albumin was detected by Human Albumin ELISA Quantitation Kits (Bethyl, USA) according to the manufacturer’s protocol.

### Statistical analyses

All statistical data reported in this article represent at least three biological replicates. P < 0.05 was considered as a significant difference between treatment groups.

## Supplementary Information

**Additional file 1: Figure S1.** DNA sequences of *FG* and *FRG* cell lines. TA clones from PCR products were analyzed by DNA sequencing. Targeted sequences are colored in blue; deletions (-). N/N indicates positive colonies out of total sequenced.

**Additional file 2: Figure S2.** Characteristics of *FG* and *FRG* pigs. (a) The birth weight of wild-type, *FG* and *FRG* pigs. (b) The survival times of wild type, *FG* and *FRG* models. (c) HE staining of liver and kidney tissues of *FG*/*FRG* and wild-type pigs at the same age. (d) The appearance of the thymus of wild type, *FG*, and *FRG* models. (e) Immunohistochemical staining of *FRG* pig liver and kidney tissues with *FAH* antibodies. Scale bar = 100 μm. (f) Transcriptomic analysis of the *FRG* pig liver tissue. Orange boxes indicate up-regulated genes, blue boxes indicate down-regulated genes.

**Additional file 3: Table S1.** Blood biochemical examination between WT and KO pigs. **Table S2.** Blood routine examination between WT and KO pigs. **Table S3.** Primer sequences for identify PCR. **Table S4.** The background information of hepatocyte donors.

## Data Availability

All data analyzed during this study are included in this published article and its supplementary information files.

## Abbreviations

Cas9: CRISPR-associated protein 9
FAH: Fumarylacetoacetate hydrolase
IL2RG: Interleukin 2 receptor γ
KO: Knockout
NTBC: 2-(2-Nitro-4-trifluoromethylbenzoyl)-1,3-cyclohexanedione
RAG1: Recombination activating gene 1
SCNT: Somatic-cell nuclear transfer
sgRNA: Single-guide RNA
