# Supplementary material for: Generation of immunodeficient pig with hereditary tyrosinemia type 1 and their preliminary application for humanized liver
Source: Cell Biosci. 2022 Mar 7;12:26. doi: 10.1186/s13578-022-00760-3 (PMC8900390; doi:10.1186/s13578-022-00760-3)
Supplement: Supplementary file 1 — Additional file 1: Figure S1. DNA sequences of FG and FRG cell lines. TA clones from PCR products were analyzed by DNA sequencing. Targeted sequences are colored in blue; deletions (-). N/N indicates positive colonies out of total sequenced. [file 13578_2022_760_MOESM1_ESM.pdf]

# Figure S1

**a**

**Cell line FG7-2**

```
FAH    WT 5'-CCCTGTGGCCGAGGATTCTGACTTCCCTATCCACAACCTGCCCTACGGCGTCTTCTCCACTACAG-3'
       KO 5'-CCCTGT----------------------------------------GGCGTCTTCTCCACTACAG-3'      Δ40 bp
IL2RG  WT 5'-ATGTTGAAGCCACCATTGCCAGTCAAATCCCTCTTATTCCTGCAGCTGCC-3'
       KO 5'-ATGTTGAAGCCACCATTGCCAGTCAAATCCCTCT...139bp...TATTCCTGCAGCTGCC-3'      +139 bp
```

**Cell line FG-2**

```
FAH    WT 5'-CCCTGTGGCCGAGGATTCTGACTTCCCTATCCACAACCTGCCCTACGGCGTCTTCTCCACTACAG-3'
       KO 5'-CCCTGT----------------------------------------GGCGTCTTCTCCACTACAG-3'      Δ40 bp
IL2RG  WT 5'-ATGTTGAAGCCACCATTGCCAGTCAAATCCCTCTTATTCCTGCAGCTGCC-3'
       KO 5'-ATGTTGAAGCCACCATTGCCAGTCAAATCCCTC----TCCTGCAGCTGCC-3'                  Δ4 bp
```

**Cell line FG-27**

```
FAH    WT 5'-CCCTGTGGCCGAGGATTCTGACTTCCCTATCCACAACCTGCCCTACGGCGTCTTCTCCACTACAG-3'
       KO 5'-CCCTGT----------------------------------------GGCGTCTTCTCCACTACAG-3'      Δ40 bp
IL2RG  WT 5'-ATGTTGAAGCCACCATTGCCAGTCAAATCCCTCTTATTCCTGCAGCTGCC-3'
       KO 5'-ATGTTGAAGCCACCATTGCCAGTCAAATCCCTC----------AGCTGCC-3'                  Δ10 bp
```

**Cell line FG-43**

```
FAH    WT 5'-CCCTGTGGCCGAGGATTCTGACTTCCCTATCCACAACCTGCCCTACGGCGTCTTCTCCACTACAG-3'
       KO 5'-CCCTGT----------------------------------------GGCGTCTTCTCCACTACAG-3'      Δ40 bp
IL2RG  WT 5'-ATGTTGAAGCCACCATTGCCAGTCAAATCCCTCTTATTCCTGCAGCTGCC-3'
       KO 5'-ATGTTGAAGCCACCATTGCCAGTCAAATCCCTC------------------3'                  Δ110 bp
```

**b**

**Cell line FRG-9**

```
FAH    WT 5'-CCCTGTGGCCGAGGATTCTGACTTCCCTATCCACAACCTGCCCTACGGCGTCTTCTCCACTACAG-3'
       KO 5'-CCCTGT----------------------------------------GGCGTCTTCTCCACTACAG-3'      Δ40 bp
RAG1   WT 5'-GCCAAAGTTTTCCGGATCGATGTG...77bp...CCCCATGTGAGGTTTACTCCCCA-3'
       KO 5'-GCCAAAGTTTTCC-----------...77bp...----------GGTTTACTCCCCA-3'           Δ100 bp
          5'-GCCAAAGTTTTCCGGATCGATGTG...77bp...CCC-ATGTGAGGTTTACTCCCCA-3'           Δ1 bp
IL2RG  WT 5'-ATGTTGAAGCCACCATTGCCAGTCAAATCCCTCTTATTCCTGCAGCTGCC-3'
       KO 5'-ATGTTGAAGCCACCATTGCCAGTCAAATCC---------------------3'                  Δ139 bp
```

**Cell line FRG-15**

```
FAH    WT 5'-CCCTGTGGCCGAGGATTCTGACTTCCCTATCCACAACCTGCCCTACGGCGTCTTCTCCACTACAG-3'
       KO 5'-CCCTGT----------------------------------------GGCGTCTTCTCCACTACAG-3'      Δ40 bp
RAG1   WT 5'-TTTAGCAGCACCCCATGTGAGGTTTACTCCCCAAGGAATGCAGCCATGGAGTGGCACCCCCACACC-3'
       KO 5'-TTTAGCAGCACCCCATG-GAGGTTTACTCCCCAAGGAATGCAGCCATGGAGTGGCACCCCCACACC-3'    Δ1 bp
          5'-TTTAGCAGCACCCCAT----GGTTTACTCCCCAAGGAATGCAGCCATGGAGTGGCACCCCCACACC-3'    Δ4 bp
IL2RG  WT 5'-ATGTTGAAGCCACCATTGCCAGTCAAATCCCTCTTATTCCTGCAGCTGCC-3'
       KO 5'-ATGTTGAAGCCACCATTGCCAGTCAAATCCCTCTTTATTCCTGCAGCTGCC-3'                 +1 bp
```

**Cell line FRG-21**

```
FAH    WT 5'-CCCTGTGGCCGAGGATTCTGACTTCCCTATCCACAACCTGCCCTACGGCGTCTTCTCCACTACAG-3'
       KO 5'-CCCTGT----------------------------------------GGCGTCTTCTCCACTACAG-3'      Δ40 bp
RAG1   WT 5'-TTTAGCAGCACCCCATGTGAGGTTTACTCCCCAAGGAATGCAGCCATGGAGTGGCACCCCCACACC-3'
       KO 5'-TTTAGCAGCACCCCATGTTGAGGTTTACTCCCCAAGGAATGCAGCCATGGAGTGGCACCCCCACACC-3'   +1 bp
          5'-TTTAGCAGCACCCCAT-----------------AAGGAATGCAGCCATGGAGTGGCACCCCCACACC-3'   Δ17 bp
IL2RG  WT 5'-ATGTTGAAGCCACCATTGCCAGTCAAATCCCTCTTATTCCTGCAGCTGCC-3'
       KO 5'-ATGTTGAAGCCACCATTGCCAGTCAAATCCCTC--ATTCCTGCAGCTGCC-3'                  Δ2 bp
```

**Cell line FRG-27**

```
FAH    WT 5'-CCCTGTGGCCGAGGATTCTGACTTCCCTATCCACAACCTGCCCTACGGCGTCTTCTCCACTACAG-3'
       KO 5'-CCCTGT----------------------------------------GGCGTCTTCTCCACTACAG-3'      Δ40 bp
RAG1   WT 5'-TTTAGCAGCACCCCATGTGAGGTTTACTCCCCAAGGAATGCAGCCATGGAGTGGCACCCCCACACC-3'
       KO 5'------------------TGAGGTTTACTCCCCAAGGAATGCAGCCATGGAGTGGCACCCCCACACC-3'    Δ17 bp
          5'-TTTAGCAGCACCC-----GAGGTTTACTCCCCAAGGAATGCAGCCATGGAGTGGCACCCCCACACC-3'    Δ5 bp
IL2RG  WT 5'-ATGTTGAAGCCACCATTGCCAGTCAAATCCCTCTTATTCCTGCAGCTGCC-3'
       KO 5'-ATGTTGAAGCCACCATTGCCAGTCA--------------------------3'                  Δ41 bp
```
