# Supplementary figures and images for: Generation of immunodeficient pig with hereditary tyrosinemia type 1 and their preliminary application for humanized liver
Source: Cell Biosci. 2022 Mar 7;12:26. doi: 10.1186/s13578-022-00760-3 (PMC8900390; doi:10.1186/s13578-022-00760-3)

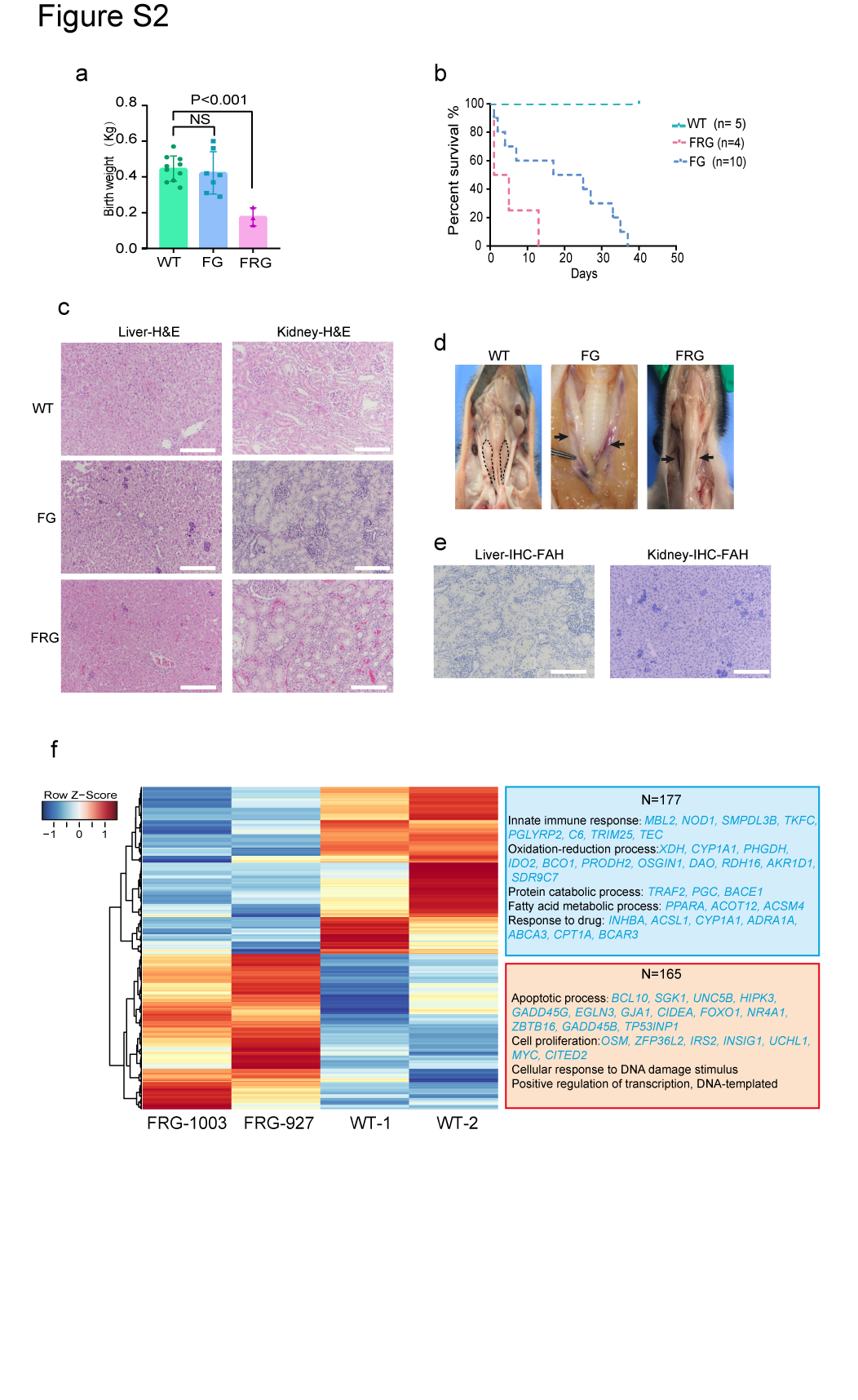

Supplement: Supplementary file 2 — Additional file 2: Figure S2. Characteristics of FG and FRG pigs. (a) The birth weight of wild-type, FG and FRG pigs. (b) The survival times of wild type, FG and FRG models. (c) HE staining of liver and kidney tissues of FG/FRG and wild-type pigs at the same age. (d) The appearance of the thymus of wild type, FG, and FRG models. (e) Immunohistochemical staining of FRG pig liver and kidney tissues with FAH antibodies. Scale bar = 100 μm. (f) Transcriptomic analysis of the FRG pig liver tissue. Orange boxes indicate up-regulated genes, blue boxes indicate down-regulated genes. [file 13578_2022_760_MOESM2_ESM.tif]
