# Supplementary material for: Generation of immunodeficient pig with hereditary tyrosinemia type 1 and their preliminary application for humanized liver
Source: Cell Biosci. 2022 Mar 7;12:26. doi: 10.1186/s13578-022-00760-3 (PMC8900390; doi:10.1186/s13578-022-00760-3)
Supplement: Supplementary file 3 — Additional file 3: Table S1. Blood biochemical examination between WT and KO pigs. Table S2. Blood routine examination between WT and KO pigs. Table S3. Primer sequences for identify PCR. Table S4. The background information of hepatocyte donors. [file 13578_2022_760_MOESM3_ESM.doc]

Table S1. Blood biochemical examination between WT and KO pigs

|  |  | WT | | | | FG | | | | FRG | |
| --- | --- | --- | --- | --- | --- | --- | --- | --- | --- | --- | --- |
| Items |  | n. | Mean±s.e.m. | | n. | | Mean±s.e.m. | n. | | | Mean±s.e.m. |
| TBIL(μmol/L) |  | 4 | 3.94±0.55 | 4 | | | 2.74±0.36 | | 2 | | 1.15±0.81* |
| DBIL(μmol/L) |  | 4 | 1.30±0.59 | 4 | | | 1.36±0.25 | | 2 | | 0.45±0.32 |
| TP(g/L) |  | 4 | 57.66±0.69 | 4 | | | 26.29±3.50** | | 2 | | 25.15±17.78** |
| ALB(g/L) |  | 4 | 42.34±1.31 | 4 | | | 8.32±0.99** | | 2 | | 9.50±6.72** |
| Glob(g/L) |  | 4 | 15.33±1.56 | 4 | | | 17.97±2.95 | | 2 | | 20.80±14.71 |
| A/G |  | 4 | 2.86±0.32 | 4 | | | 0.49±0.06** | | 2 | | 0.70±0.49* |
| GLU(mmol/L) |  | 4 | 10.13±2.02 | 4 | | | 10.19±6.04 | | 2 | | 0.71±0.50* |
| ALT(U/L) |  | 4 | 38.34±5.29 | 4 | | | 41.35±14.94 | | 2 | | 69.00±48.79 |
| AST(U/L) |  | 4 | 72.55±8.06 | 4 | | | 122.25±34.98 | | 2 | | 83.00±58.69 |
| GGT(U/L) |  | 4 | 38.17±0.95 | 4 | | | 59.45±19.19 | | 2 | | 82.50±58.34 |
| ALP(U/L) |  | 4 | 1875.06±146.19 | 4 | | | 2362.70±605.26 | | 2 | | 1410.00±997.02 |
| TBA(μmol/L) |  | 4 | 8.98±0.64 | 4 | | | 14.80±3.10 | | 2 | | 9.30±6.58 |
| CRE(μmol/L) |  | 4 | 37.28±3.35 | 4 | | | 94.99±19.55* | | 2 | | 87.10±61.59* |
| UREE(mmol/L) |  | 4 | 5.96±0.50 | 4 | | | 4.28±0.33* | | 2 | | 14.27±10.09 |
| Ca(mmol/L) |  | 4 | 3.17±0.03 | 4 | | | 3.10±0.37 | | 2 | | 3.28±2.32 |
| P(mmol/L) |  | 4 | 3.70±0.19 | 4 | | | 3.16±0.75 | | 2 | | 4.77±3.37 |
| CHO(mmol/L) |  | 4 | 12.19±1.22 | 4 | | | 1.11±0.33** | | 2 | | 1.87±1.32** |
| TG(mmol/L) |  | 4 | 1.77±0.37 | 4 | | | 0.55±0.26* | | 2 | | 0.20±0.14* |
| AMY(U/L) |  | 4 | 1410.71±202.17 | 4 | | | 736.80±85.76* | | 2 | | --- |
| CK(U/L) |  | 4 | 1980.50±591.34 | 4 | | | 2195.50±289.79 | | 2 | | 2720.50±1923.68 |
| LDH(U/L) |  | 4 | 2185.27±177.01 | 4 | | | 1580.60±169.71* | | 2 | | 1419.50±1003.74* |

Quantitative data are presented as mean±s.e.m. Significance was established using a 2-tailed Student’s t-test. Differences were considered significant at *P<0.05, **P<0.01.

Table S2 Blood routine examination between WT and KO pigs

|  |  | WT | | | FG | | | FRG | |
| --- | --- | --- | --- | --- | --- | --- | --- | --- | --- |
| Items |  | n. | Mean±s.e.m. | n. | | Mean±s.e.m. | n. | | Value. |
| RBC(×10^12^/L) |  | 4 | 5.29±0.23 | 4 | | 4.24±0.59 | 1 | | 2.31 |
| HGB(g/L) |  | 4 | 107.33±3.53 | 4 | | 79.50±10.87 | 1 | | 41.00 |
| HCT(%) |  | 4 | 36.60±1.40 | 4 | | 27.58±3.99 | 1 | | 14.50 |
| MCV(fL) |  | 4 | 69.43±0.62 | 4 | | 64.93±1.57 | 1 | | 62.70 |
| MCH(pg) |  | 4 | 20.37±0.30 | 4 | | 18.75±0.27* | 1 | | 17.50 |
| MCHC(g/L) |  | 4 | 293.67±1.76 | 4 | | 289.50±6.14 | 1 | | 279.00 |
| PLT(×10^9^/L) |  | 4 | 523.67±117.36 | 4 | | 190.25±46.59* | 1 | | 30.00 |
| WBC(×10^9^/L) |  | 4 | 9.07±1.56 | 4 | | 2.75±1.20* | 1 | | 3.83 |
| SEG% |  | 4 | 33.93±2.90 | 4 | | 46.83±15.34 | 1 | | --- |
| BAND% |  | 4 | 0.37±0.12 | 4 | | 0.15±0.06 | 1 | | --- |
| MON% |  | 4 | 2.33±0.37 | 4 | | 7.53±5.15 | 1 | | 35.20 |
| LYM% |  | 4 | 62.67±2.84 | 4 | | 39.35±7.61** | 1 | | 5.80 |
| EOS% |  | 4 | 1.00±0.35 | 4 | | 1.78±0.84 | 1 | | 0.00 |
| BAS% |  | 4 | 0.07±0.03 | 4 | | 4.53±2.65 | 1 | | 2.50 |

Quantitative data are presented as mean±s.e.m(except for FRG pigs). Significance was established using a 2-tailed Student’s t-test. Differences were considered significant at *P<0.05, **P<0.01.

Table S3 Primer sequences for identify PCR

| Primer name | Sequences (5`-3`) | Product length |
| --- | --- | --- |
| JD-FAH-F | CTGCTGGGTCAAGGGGTCAACTCTG | 299bp |
| JD-FAH-R | ggggttctgggcgaagggga |
| JD-RAG1-F | TCCCACGAAGATGGGAAAGC | 513bp |
| JD-RAG1-R | TTCCTTGCTGCTGATCCTGG |
| JD-IL2RG-F | CAGACCCTCCACTACATGGG | 673bp |
| JD-IL2RG-R | AGAAGAAGCAGCACTAGGCA |

Table S4 The background information of hepatocyte donors

| Lot Number | Age | Gender | Race | Cause of Death |
| --- | --- | --- | --- | --- |
| MRW*** | 11months | M | C | Asphyxiation |
| FCL | 10 months | F | H | Anoxia/drowning |
